# The effectiveness of low-frequency electrical stimulation in treating hemiplegic shoulder pain: a systematic review and meta-analysis

**DOI:** 10.3389/fneur.2025.1574338

**Published:** 2025-05-30

**Authors:** Tao Qin, Tiantian Hu, Yuzhuo Dan, Cheng Qiu, Mei Chen, Fanjing Kong, Sha Huang, Zhenwei Zhai, Ying Xu, Tao Sun

**Affiliations:** ^1^Hospital of Chengdu University of Traditional Chinese Medicine, Chengdu, China; ^2^Department of Rehabilitation Medicine, The General Hospital of Western Theater Command, Sichuan, Chengdu, China; ^3^School of Intelligent Medicine, Chengdu University of Traditional Chinese Medicine, Chengdu, China; ^4^The Acupuncture and Tuina School, Chengdu University of Traditional Chinese Medicine, Chengdu, China; ^5^State Key Laboratory of Southwestern Chinese Medicine Resources, Chengdu University of Traditional Chinese Medicine, Chengdu, China

**Keywords:** hemiplegic shoulder pain, low-frequency electrical stimulation, stroke, systematic review, meta-analysis

## Abstract

**Objective:**

To clarify the effectiveness of Low-frequency electrical stimulation (LFES) in treating Hemiplegic shoulder pain (HSP), identify the therapeutic effects of different treatment parameters, and provide evidence-based recommendations.

**Methods:**

We searched PubMed, EMBASE, Web of Science, Cochrane Library, China National Knowledge Infrastructure, Wanfang Data, and China Science and Technology Journal Database up to October 2023. Randomized controlled trials (RCTs) comparing LFES with comparable single rehabilitation interventions, placebo/sham treatments, or conventional rehabilitation were included. The included outcomes were pain intensity and motor function in the upper extremities. The systematic review protocol is available on the International Prospective Register of Systematic Reviews (PROSPERO) (registration number: CRD42023493979).

**Results:**

A total of eight studies (341 participants) were included. LFES showed significant therapeutic effects on shoulder pain scores (SMD = −0.68; 95% CI: [−1.18, −0.18], *Z* = 2.69, *p* = 0.006, *I*^2^ = 76%). However, the improvement in upper limb motor function (MD = 8.50; 95% CI: [5.12, 11.88], *Z* = 4.93, *p* < 0.001, *I*^2^ = 16%) was influenced by a single study with lower methodological quality. Subgroup analyses examined factors such as control group type, stimulation type, duration, frequency, pulse width, and stimulation area. The variations in therapeutic effects of LFES across different treatment parameters, different hemiplegic sides, and different stimulation areas were further explored by subgroup analysis.

**Conclusion:**

The meta-analysis results indicate that LFES has significant positive effects on alleviating HSP, but its effect on improving motor function requires cautious interpretation due to potential bias.

**Systematic review registration:**

International Prospective Register of Systematic Reviews (PROSPERO), identifier: CRD42023493979.

## Introduction

Stroke is a significant public health issue. According to the results of the Global Burden of Diseases 2019, stroke remains the second leading cause of death worldwide, third for disability, and the primary cause of dementia (1). It is projected that by 2050, the absolute number of global deaths due to stroke will increase to about 9.7 million (6.6 million, 2020), with a sharp increase in the proportion coming from low-income and lower-middle-income countries (approximately 109.95 per 100,000 people) relative to 2020 (2). Currently, there is a regional distribution in the incidence rates, especially in low-income countries (accounting for 83.7% of the global incidence of the disease) and lower-middle-income countries (accounting for 87.6% of the global incidence) (1). In the United States, about 0.8 million people suffer from it annually (3); in Europe, about 1.1 million residents are affected each year, with the economic loss related to this amounting to approximately 45 billion euros in 2017 (4); in 2020, the number of patients in Asia was about 4.1 million, accounting for 61.3% of the global total of stroke patients. Thus, this disease poses serious survival issues and economic burdens globally.

Various functional impairments caused by stroke, such as hemiplegia (5), hemiplegic shoulder pain (HSP) (6), spasticity (7), dysphagia (8), and speech and cognitive disorders (9), affect their life cycle and quality of life to varying degrees. Among these, HSP shows a more severe short-term prognosis compared to other complications caused by stroke, affecting the recovery of upper limb motor function, prolonging the Hospitalization period, shortening their life cycle, increasing the risk of disability, and tendency toward depression (10, 11). In the first week, the prevalence of HSP among stroke patients is 17%, increasing to 22–40% in 4–6 months (12). Given the significant impact of HSP on stroke patients, especially in terms of disability and depression, its prevalence and severity have received widespread attention in the academic field in recent years (13). However, the etiopathogenesis of HSP has not yet been fully elucidated. Current research primarily focuses on musculoskeletal issues such as shoulder subluxation, subacromial-deltoid bursitis, and spasticity (14), while neurological factors like neuropathic pain and central sensitization are increasingly recognized (15). Researchers are exploring these mechanisms and developing interventions to reduce HSP’s impact on outcomes.

Common treatments for HSP include physical therapy such as electrical stimulation (ES), sling (16), Soft Tissue Massage (17), magnetic stimulation (18), rehabilitation robots (19), as well as drug injections (20), surgical treatment, and various therapeutic approaches combined with AI technology (21). Among these treatments, soft tissue massage and shoulder slings are commonly used as conservative treatments to manage pain and improve mobility in HSP patients. However, their long-term efficacy varies. Magnetic stimulation and rehabilitation robots remain in early research stages, with limited high-quality evidence for their long-term efficacy (22, 23). Similarly, drug injections and surgical treatments show inconsistent effectiveness and are generally used when conservative approaches fail (24, 25). In contrast, ES is widely used in clinical practice due to its higher targeted effectiveness, better patient compliance, and fewer side effects. In the treatment of ES, low-frequency electrical stimulation (LFES) is more commonly applied. Although there are many types of LFES, their analgesic principles in HSP can mainly be divided into two types. Specifically, for pain caused by myogenic factors, LFES can relieve pain by inducing muscle contractions (26); it can also target pain relief at the sensory level without causing any muscle contraction, so it is sometimes preferred for treating neuropathic pain (27).

In clinical practice, the main efficacy indicators for evaluating HSP are pain-related indicators. However, the overall efficacy of ES in reducing shoulder pain remains debated. While some studies suggest potential benefits of ES in mitigating shoulder subluxation and improving range of motion (28), the evidence on its direct impact on pain relief is inconsistent (29–31). This disparity highlights the need for further research to determine the specific conditions in which ES offers the greatest benefit for individuals with HSP. At the same time, we find that in some randomized controlled trials (RCTs), the series of parameters of LFES treatment for HSP have different impacts on outcome indicators. Existing discussions on the selection of types, parameters, and placement of treatment electrodes for LFES to treat HSP with the expectation of better prognosis are scarce. In addition, we also notice that some review articles clearly indicate that a single type of LFES has a certain therapeutic effect on HSP (29, 32, 33), but a comprehensive comparison of more types of LFES has not been fully considered. Therefore, more systematic research is needed on LFES for treating HSP to clarify the best treatment parameters and types.

Therefore, based on this highly discussed issue, our study aims to investigate the therapeutic effects of LFES in HSP and explore the differences in treatment efficacy under various conditions, including different treatment parameters (stimulation type, stimulation frequency, pulse width, stimulation duration, etc.), different hemiplegic sides, and different stimulation areas, to provide optimized references for the formulation of treatment plans.

## Method

### Study design

This systematic review with meta-analysis was conducted according to the Preferred Reporting Items for Systematic Reviews and Meta-analysis (PRISMA) guidelines and the Cochrane Handbook for Systematic Reviews of Interventions (34). The systematic review protocol is available on the International Prospective Register of Systematic Reviews (PROSPERO) (registration number: CRD42023493979).

### Search strategy

We systematically searched several electronic databases, including PubMed (MEDLINE), Excerpta Medica Database; ES: electrical stimulation (EMBASE), Web of Science, Cochrane Library, China National Knowledge Infrastructure (CNKI), Wanfang Data, China Science and Technology Journal Database (VIP) for articles published from inception to October 24, 2023, according to each specific thesaurus. No language or country limitations were applied. The following combination of search keywords was used: (“electrical stimulation” OR “low-frequency electrical stimulation” OR “transcutaneous electrical nerve stimulation” OR “biofeedback electrical stimulation” OR “neuromuscular electrical stimulation” OR “muscle electrical stimulation” OR “functional electrical stimulation” OR “peripheral nerve stimulation” OR “percutaneous nerve stimulation” OR “microcurrent stimulation” OR “TENS” OR “FES” OR “NMES” OR “PNS” OR “LFES”) AND (“stroke shoulder pain” OR “hemiplegic shoulder pain” OR “painful hemiplegic shoulder”). The detailed search strategy used is described in Supplementary Table S1.

### Inclusion and exclusion criteria

Our inclusion criteria were based on the PICO framework:

- P) Participants: stroke survivors with HSP.
- I) Intervention: LFES, including neuromuscular electrical stimulation (NMES), transcutaneous electrical nerve stimulation (TENS), biofeedback electrical stimulation, muscle electrical stimulation, functional electrical stimulation (FES), peripheral nerve stimulation (PNS), percutaneous nerve stimulation and microcurrent stimulation.
- C) Comparator: comparable single rehabilitation interventions, placebo/sham treatments, and conventional rehabilitation.
- O) Outcome measure: the primary outcome in the study is pain intensity, using a visual analogue scale (VAS) (35) or numerical rating scale (NRS) (36). The secondary outcome is the Fugl-Meyer assessment for upper extremities (FMA-UE).

We excluded studies (1) not RCTs; (2) including Children or growing participants; (3) focusing on other complications associated with HSP (e.g., glenohumeral subluxation); (4) causing shoulder pain due to other factors (e.g., head injury, shoulder subluxation); (5) with no full text (e.g., posters and conference abstracts); (6) unpublished or retracted; (7) *in vitro* or on animal experiments and (8) with no apparent sample size.

The decision to designate pain intensity as the primary outcome in this study is based on several key considerations. First, HSP is one of the most common and disabling complications following stroke, with pain intensity not only directly reflecting the patient’s subjective suffering but also closely associated with decreased quality of life, sleep disturbances, and reduced participation in rehabilitation (37, 38). Therefore, selecting pain intensity as the primary outcome allows for quantification of the patient’s most prominent subjective symptom, which in turn facilitates prediction and improvement of quality of life, rehabilitation engagement, and functional recovery. Second, accurate measurement of pain intensity is fundamental to clinical intervention and essential for evaluating treatment efficacy. The VAS and NRS are the primary tools used to assess pain intensity; these instruments are widely applied in HSP research due to their high reproducibility and excellent psychometric properties (39). Moreover, they are endorsed by the internationally recognized IMMPACT guidelines (Initiative on Methods, Measurement, and Pain Assessment in Clinical Trials) as gold standards for chronic pain assessment, ensuring scientific rigor and comparability across studies (14). Finally, although upper limb motor function measures such as the FMA-UE serve as important secondary outcomes, substantial empirical evidence indicates that pain relief often precedes and enables functional recovery (40). Unresolved pain can hinder patients’ adherence to rehabilitation exercises, whereas effective pain reduction significantly enhances engagement and therapeutic outcomes. Thus, prioritizing pain intensity as the primary outcome not only focuses on the patient’s most immediate subjective experience but also provides a critical evaluation metric for personalized rehabilitation strategies.

### Data collection

Two independent investigators (YD and CQ) were in charge of study selection and data extraction, while a third investigator participated in discussions and made decisions regarding discrepancies. Study titles and abstracts were reviewed for eligibility, and full texts were retrieved for further screening.

The following data were extracted from each selected study: (1) first author, (2) publication year, (3) participant count, (4) participant age, (5) duration after stroke, (6) treatment duration and length of follow-up, (7) side of stroke, and (8) outcome measures (mean and standard deviation) such as pain intensity (VAS or NRS) and FMA-UE at the end of treatment for both the experimental and control groups. We extracted data from published articles by reading the full text and searching clinicaltrials.gov using identified NCT numbers for additional information. If necessary, we also contacted the study authors.

For multiple post-treatment measures, only end-of-treatment data were used. When multiple independent groups were included in one study (e.g., different stimulation types), these data were extracted and treated as an intervention group, and the corresponding control group was also treated separately without duplicating participants.

### Evaluation of the methodology of the studies selected (risk of bias)

The extracted data from the selected studies were synthesized. Two authors (TQ and TH) independently assessed the quality of the RCT reports using the Physiotherapy Evidence Database Scale (PEDro) (41). In case of disagreement, a third author was consulted to reach a consensus. We searched the included RCTs in the PEDro database.^1^ Additionally, we manually assessed all included studies using the PEDro criteria to verify the reliability of the database ratings. According to the PEDro scale 32, the studies were meta-analysis categorized as excellent (9–10 points), good (6–8 points), fair (4–5 points), or poor (<4 points). Additionally, two authors evaluated the risk of bias in RCTs using Version 2 of the Cochrane Risk of Bias tool for randomized trials (RoB 2) (42). The PEDro scale is widely regarded as the gold standard for assessing methodological quality in rehabilitation research. However, it may overlook certain biases such as selection bias and performance bias (41). Conversely, while the Cochrane RoB 2 tool provides a deeper understanding of potential biases, it is not tailored to the rehabilitation field and may neglect methodological issues specific to physical therapy (42). By integrating these two tools in our quality assessment, a more comprehensive and reliable evaluation might be achieved, thereby enhancing the quality and credibility of evidence interpretation. Any differences in opinion were discussed with a third author. The Grading of Recommendations Assessment, Development and Evaluation (GRADE) framework is utilized to evaluate the quality of evidence in meta-analyses and subgroup analyses. Initially, all RCTs are assigned a “high” quality rating. The rating can be downgraded by one or two steps based on five criteria, including the risk of bias in individual studies, inconsistency of study results, indirectness of evidence, imprecision, and publication bias. The final assessment of evidence quality is divided into four levels. GRADE’s approach to providing certainty ratings for each outcome in the body of evidence based on systematic reviews and across outcomes is detailed in Supplementary Figure S1.

### Statistical analysis

Meta-analysis was performed using Review Manager (RevMan) software, version 5.3, and R 4.2.1 (R; GitHub, San Francisco, US). When the same assessment scales were used, weighted mean difference (WMD) with 95% confidence interval (CI) was calculated; otherwise, standardized mean difference (SMD) was used. Heterogeneity was evaluated using *I*^2^ tests, with *I*^2^ > 50% indicating high heterogeneity (43). Fixed effects models were employed when heterogeneity findings were nonsignificant, while random effects models were utilized for high heterogeneity findings. Sensitivity analysis was performed by systematically removing individual studies one by one. To assess differences between subgroups in the meta-analysis, the overlap of 95% CIs was examined. According to the Cochrane Handbook for Systematic Reviews of Interventions, overlapping 95% CIs between subgroups suggest that there is no statistically significant difference between these subgroups. Therefore, between-subgroup differences were interpreted based on the presence or absence of overlap in their 95% CIs. This approach was applied consistently throughout the subgroup analyses (44). To mitigate the potential impact of publication bias, we assessed its presence by utilizing funnel plots (45). Egger’s test and Begg’s test (*p* < 0.05) were used to explore potential publication bias. The statistical analyses were performed using the R package “metafor.”

## Results

### Study selection

**Figure 1:**
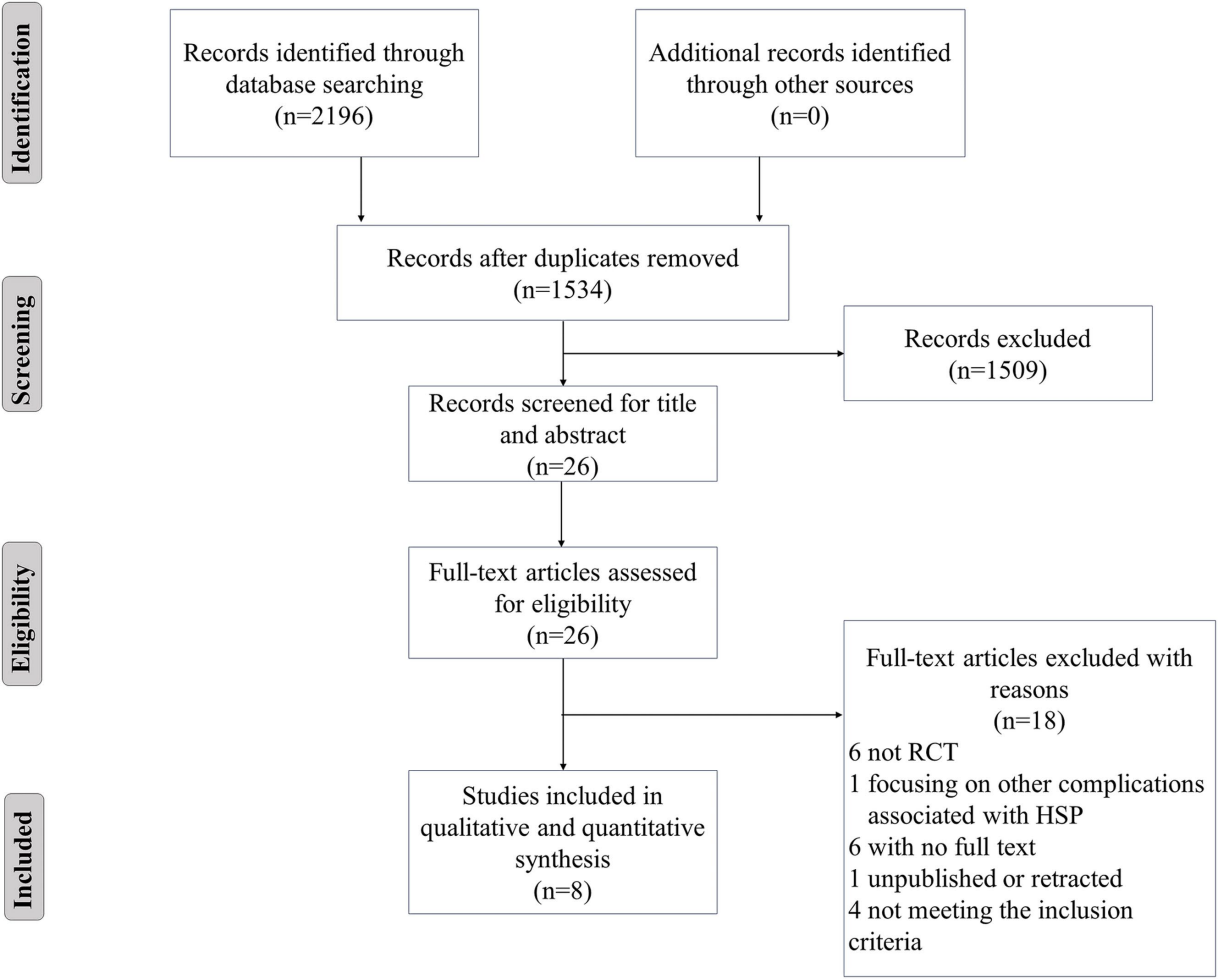
Preferred Reporting Items for Systematic Reviews and Meta-analysis (PRISMA) flow diagram of included and excluded studies.

Following the elimination of duplicate records from the initial pool of 2,196 articles identified across seven databases (Figure 1), a total of 1,534 unique articles remained for subsequent screening. A comprehensive review of the title and abstract was conducted, resulting in the exclusion of 1,509 articles. Subsequently, 26 full-text articles were identified and retrieved for further evaluation. Ultimately, a total of eight RCTs (16, 17, 46–51) (10 groups) met the inclusion criteria and were included in our systematic review. In a three-arm RCT, we divided the control group into two separate groups for analysis without duplicating data. To provide a comprehensive overview of the stimulation protocols, we have compiled detailed LFES parameters for all included studies in Supplementary Table 2.

To assess whether the inclusion of RCTs with different control groups (sham/placebo versus active treatments) affected the results of the meta-analysis, both subgroup and sensitivity analyses were conducted. In the subgroup analysis, the effects of LFES on pain were compared between studies with sham or placebo control groups and those with active treatment control groups. No statistically significant differences were observed in the change of pain scores between these two types of control groups, as indicated by partially overlapping 95% CIs (*χ*^2^ = 0.39, *p* = 0.53). In the sham/placebo group, LFES showed a significant effect in alleviating pain (SMD = −0.85, 95% CI: [−1.54, −0.16]; *Z* = 2.43, *p* = 0.02; *I*^2^ = 73%). Although LFES did not demonstrate statistical significance in improving pain scores when compared to active treatments (SMD = −0.52, 95% CI: [−1.30, 0.26]; *Z* = 1.30, *p* = 0.19; *I*^2^ = 82%), the difference between the sham or placebo group and the active treatment group did not reach statistical significance (Supplementary Figure S2). To further validate the robustness of the meta-analysis results, a sensitivity analysis was conducted by excluding RCTs with active treatment controls. The exclusion did not significantly alter the effect size or statistical significance of LFES compared to sham/placebo controls, reinforcing the reliability of the findings. This suggests that the inclusion of both types of RCTs would not compromise the overall conclusions of the meta-analysis.

### General characteristics of studies

Table 1 describes the detailed characteristics of each study included in our analysis. The eight studies included in the analysis were published between 2007 and 2022. One was conducted in Nigeria, two in the United States, two in China, and three in Turkey. We analyzed data for 341 stroke survivors (215 men and 126 women). Of these, 197 were in the experimental group, and 144 were in the control group. The sample sizes ranged from 19 to 61 patients. The mean stroke duration varied between 13.2 days and 227.2 weeks, and the mean age of the participants was between 54.0 and 69.3 years. Additionally, 53.4% of the survivors had left-sided hemiplegia, while 46.6% had right-sided hemiplegia.

**Table 1:** Characteristics of include studies.

Study	Design	Nationally	Study group	Control group	Intervention	Comparison	Outcome measure and time-point assessments	Results
Ayfle et al., 2008 (46)	2-arm RCT	Turkey	*n* = 10 sex: 6 M/4 F Age: 57.2 ± 8.7 years Side of hemiplegia: 60% R/40% L Time after stroke: 13 ± 2.4 months	*n* = 9 sex: 5 M/4 F Age: 55.9 ± 8.4 years Side of hemiplegia: 66.7% R/33.3% L Time after stroke: 12.3 ± 2.4 months	100 HZ, 200 μs, 5–9 mA TENS (20 min/d, 5/week, 3 weeks) +conventional rehabilitation	Sham TENS + conventional rehabilitation	VAS at baseline and at 3 weeks (at the end of treatment)	VAS scores were found to be lower at the end of treatment and TENS proved to be significantly effective in reducing pain (*p* < 0.05)
Badaru et al., 2020 (17)	2-arm RCT	Nigeria	*n* = 25 sex: 14 M/11 F Age: 56 ± 9.26 years Side of hemiplegia: 60% R/40% L Time after stroke: 10 ± 6 months	*n* = 25 sex: 15 M/10 F Age: 57 ± 7.51 years Side of hemiplegia: 44% R/56% L Time after stroke: 9 ± 4 months	80 HZ, 60 μs, TENS (30 min/d, 2/week, 8 weeks)	Soft tissue massage	VAS at baseline and at 8 weeks (at the end of treatment)	VAS scores were found to be lower at the end of treatment and TENS proved to be significantly effective in reducing pain (*p* < 0.05)
John et al., 2007 (16)	4-arm RCT	United states	**EARLY** *n* = 16 sex: 10 M/6 F Age: 61.6 (11.3) years Side of hemiplegia: 43.7% R/56.3% L Time after stroke: 35.4 (16.4) weeks **LATE** *n* = 16 sex: 8 M/8 F Age: 57.1 (11.9) years Side of hemiplegia: 25% R/75% L Time after stroke: 211.4 (191.3) weeks	**EARLY** *n* = 14 sex: 9 M/5 F Age: 59.1 (13.8) years Side of hemiplegia: 64.3% R/35.7% L Time after stroke: 28.6 (14.2) weeks **LATE** *n* = 15 sex: 7 M/8 F Age: 55.6 (11.8) years Side of hemiplegia: 53.3% R/46.7% L Time after stroke: 227.2 (191.3) weeks	10–200 μs, 20 mA, intramuscular NMES (6 h/d, 7/week, 6 weeks) to the supraspinatus, posterior deltoid, middle deltoid	Wear the sling, 6 h/d, 7/week, 6 weeks	BPI 12 at baseline and EOT and at 3/6/12mouths posttreatment	**EARLY** The ES group exhibited significantly greater reduction in BPI 12 scores at all posttreatment assessments. **LATE** The ES group exhibited greater reduction in BPI 12 scores at EOT compared to controls, although the difference did not reach statistical significance. The reduction in BPI 12 scores at subsequent visits were similar between groups.
Kong et al., 2015 (49)	2-arm RCT	China	*n* = 30 sex: 18 M/12 F Age: 65.23 ± 14.09 years Side of hemiplegia: 50% R/50% L Time after stroke: 13.20 ± 8.31 days	*n* = 30 sex: 16 M/14 F Age: 65.13 ± 10.46 years Side of hemiplegia: 56.7% R/43.3% L Time after stroke: 16.53 ± 10.99 days	1–100 Hz, 50 mA FES (15 min/d, 5/week, 4 weeks) + conventional rehabilitation	conventional rehabilitation	McGill (VAS) and FMA-UE at baseline and at 4weeks (at the end of treatment)	The McGill Pain Score (VAS) of the study group was significantly lower than that of the control group after 20 treatments (*p* < 0.05).
Sedef et al., 2022 (47)	2-arm RCT	Turkey	*n* = 12 sex: 10 M/2 F Age: 62.3 ± 11.2 years Side of hemiplegia: 66.7% R/33.3% L Time after stroke: 9.5 ± 8.0 months	*n* = 12 sex: 10 M/2 F Age: 69.3 ± 8.5 years Side of hemiplegia: 25.0% R/75.0% L Time after stroke: 11.6 ± 14.6 months	100 Hz, 300 μs, 0–100 mA, TENS (30 min/d, 5/week, 3 weeks) + conventional rehabilitation	Suprascapular nerve block (SSNB) + conventional rehabilitation	VAS at baseline and at first week of treatment and at 3 weeks (at end of treatment)	VAS scores were found to be lower at 1st and 3rd weeks and TENS proved to be significantly effective in reducing pain (*p* < 0.05)
Ozgur et al., 2018 (48)	2-arm RCT	Turkey	*n* = 12 sex: 6 M/6 F Age: 56 ± 17.5 years Side of hemiplegia: 41% R/59% L Time after stroke: 46.8 ± 10.3 days	*n* = 9 sex: 7 M/2 F Age: 58 ± 15.4 years Side of hemiplegia: 67% R/33% L Time after stroke: 35.2 ± 35.7 days	20 Hz, 300 μs, FES (15 min/d, 5/week, 4 weeks) + 30 min standard rehabilitation	30 min standard rehabilitation	NRS and FMA-UE at baseline and at 4 weeks (at the end of treatment)	NRS (VAS) of the study group was significantly lower than that of the control group after 20 treatments (*p* < 0.05).
Richard et al., 2014 (50)	2-arm RCT	United states	*n* = 13 sex: 7 M/6 F Age: (median +/− IQ range) 54.0 (50.0 – 68.0) years Side of hemiplegia: 38.5% R/61.5% L Time after stroke: 2.6 (0.9 – 4.0) years	*n* = 12 sex: 7 M/ 5 F Age: (median ± IQ range) 55.5 (50.0–62.5) years Side of hemiplegia: 33.3% R/66.7% L Time after stroke: 2.3 (0.8–4.8) years	12 Hz, 20 mA, 40–200 μs PNS (6 h/d, 3 weeks, total 126 h)	Physical therapy	BPI-SF3 (NRS) at baseline, end of treatment, and 6 weeks and 12 weeks post-treatment	The pain reduction with treatment for PNS group was significantly greater than the UC group (time by group interaction effect), although both groups experienced a significant pain reduction with treatment (time effect).
Zhou et al., 2018 (51)	3-arm RCT	China	**NMES** *n* = 31 sex: 10 M/2 F Age: 59.35 ± 10.78 years Side of hemiplegia: 48.39% R/51.61% L Time after stroke: 73.61 ± 53.40 days **TENS** *n* = 32 sex: 10 M/2 F Age: 58.50 ± 9.07 years Side of hemiplegia: 59.37% R/40.63% L Time after stroke: 100.88 ± 103.32 days	*n* = 18 sex: 7 M/5 F Age: 63.78 ± 11.17 years Side of hemiplegia: 38.89% R/61.11% L Time after stroke: 105.89 ± 142.80	NMES (15 Hz, 200 μs, 20–50 mA) TENS (15 Hz, 200 μs, 20–50 mA) 1 h/d, 5/week, 4 weeks +standardized rehabilitation program	Standardized rehabilitation program	NRS and FMA at baseline and 2/4/8 weeks after treatment	NRS scores, were decreased by an average of 2.03, 1.44, and 0.61 points in NMES, TENS, and the control groups after 20 sessions. All differences were statistically significant among the 3 groups (*p* < 0.001). the efficacy of the NMES group was superior to that of the TENS group, and that of the NMES and TENS group was superior to that of the control group (*p* < 0.001)

RCT, Randomized controlled trials; TENS, Transcutaneous electrical nerve stimulation; VAS, Visual Analogue Scale; NMES, Visual Analogue Scale; EOT, End of treatment; ES, Electrical stimulation; FES, Functional electrical stimulation; FMA-UE, Fugl-Meyer assessment for upper extremities; PNS, Percutaneous nerve stimulation; UC, Usual care; NRS, Numerical rating scale.

### Risk of bias

**Figure 2:**
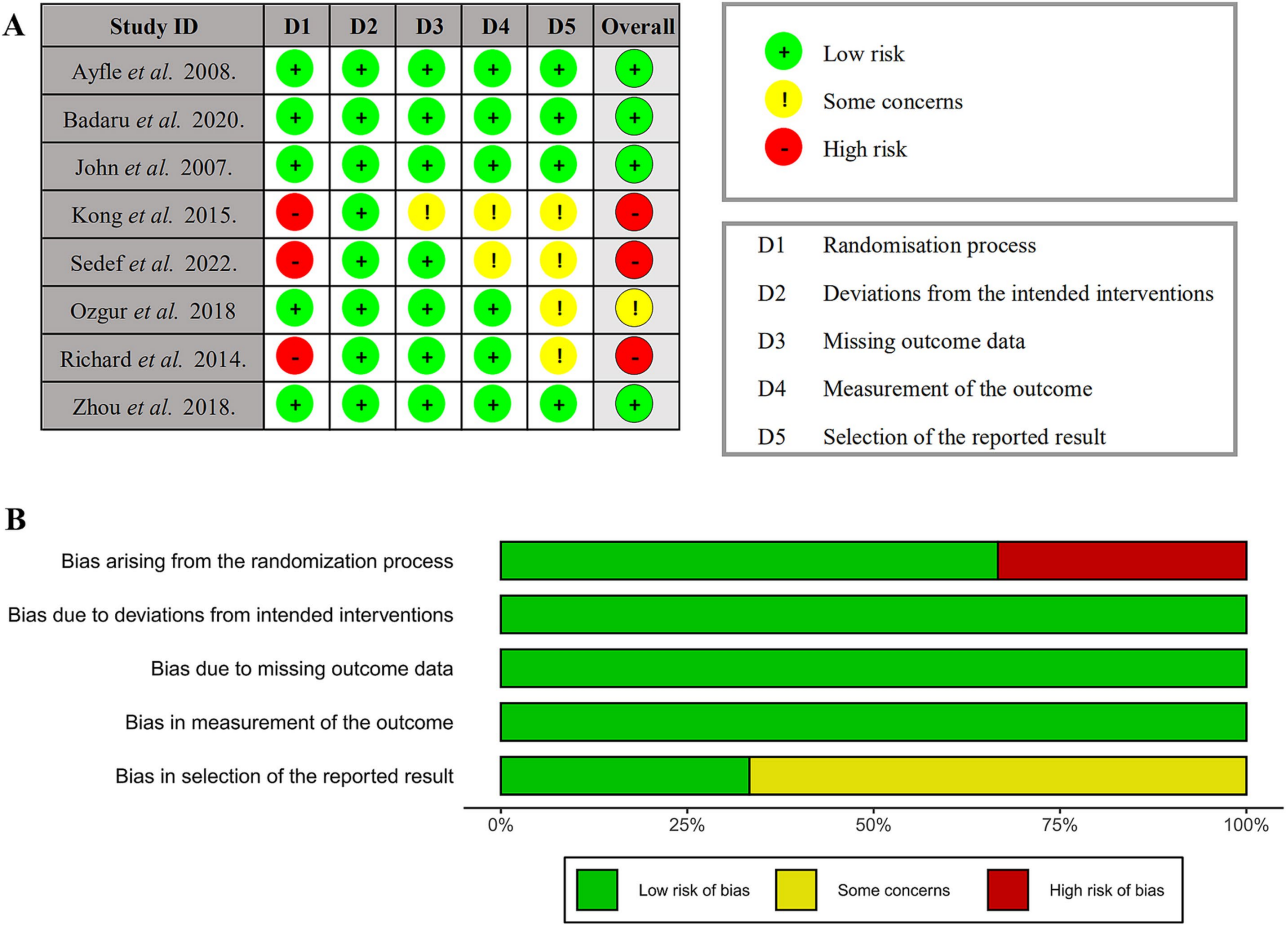
Cochrane risk of bias tool for randomized trials Version 2 (RoB 2.0). **(A)** Risk of bias graph. **(B)** Risk of bias summary.

**Figure 3:**
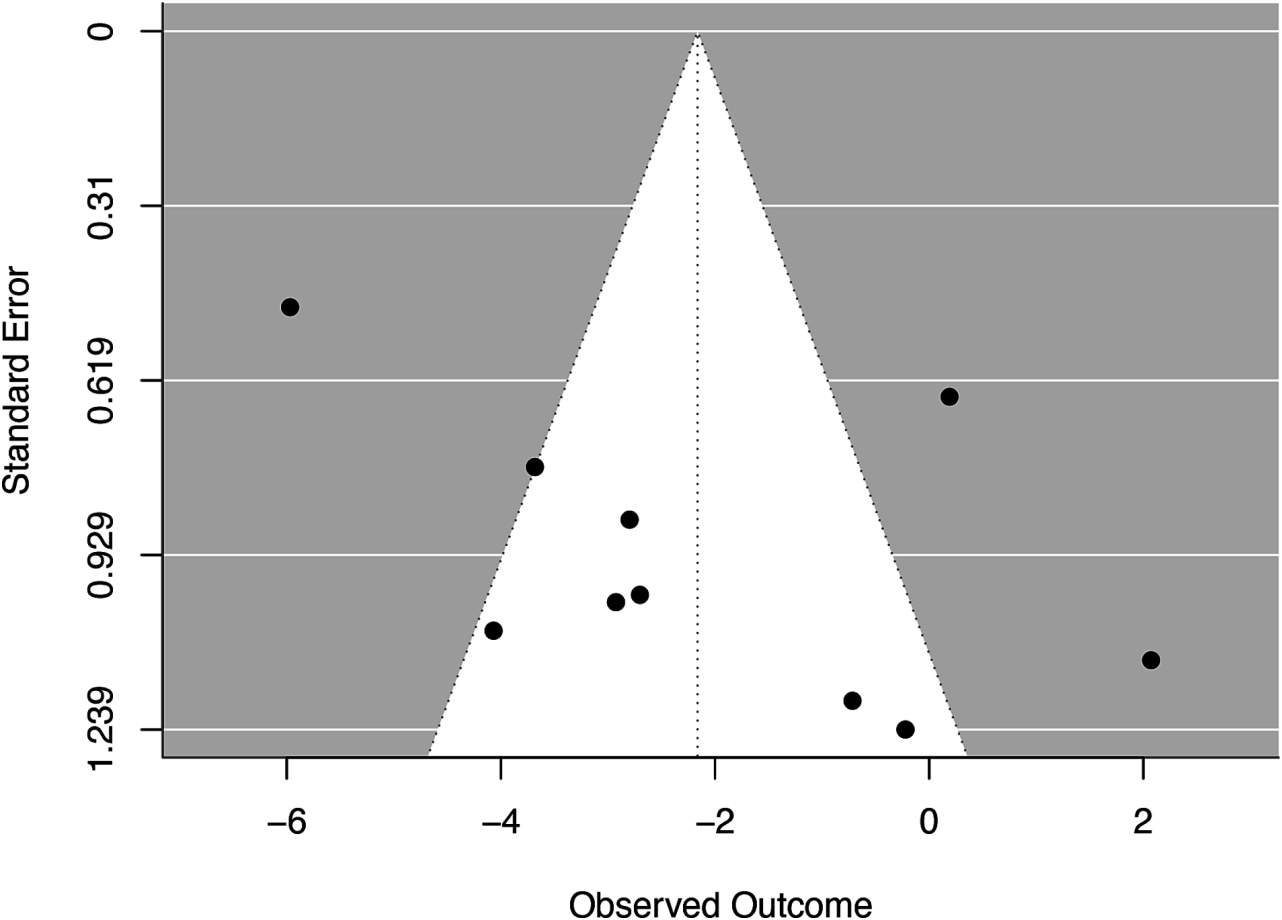
Funnel plot for observed outcome.

Among the eight RCTs studies conducted, four reported the application of a blinding method, four reported the post-treatment follow-up period, and five reported allocation concealment. According to the PEDro bias scale, six studies were rated as having “good” methodological quality (6–8 scores). The remaining two studies were rated as having “fair” methodological quality (4–5 scores). The results of the quality assessment are presented in Table 2. Two authors (TQ and TH) independently assessed the potential risk of bias in all included studies using RoB 2. Figure 2 presents the estimation of the risk of bias among the analyzed RCTs. The corresponding funnel plot (Figure 3) shows no asymmetry between the comparison groups of the pain scale improvement regarding WMDs. Publication bias was formally assessed using both Egger’s linear regression test (*Z* = 1.68, *p* = 0.093) and Begg’s rank correlation test (Kendall’s *τ* = 0.29, *p* = 0.29). Although no statistically significant bias was detected, the observed heterogeneity in pain outcomes (*I*^2^ = 87.31%) suggests the need for cautious interpretation of these results. The GRADE evidence quality evaluation of meta-analysis and subgroup analysis are presented in Table 3.

**Table 2:** Quality assessment of the included studies according to the PEDro scale.

Studies	Eligibility	Randomized allocation	Concealed allocation	Baseline comparability	Blinding of subjects	Blinding of therapists	Blinding of assessors	Key outcomes	Intention to treat	Between group comparison	Measures of variability	PEDro scale
Badaru (17)	Yes	Yes	Yes	Yes	No	No	Yes	Yes	No	Yes	Yes	7/10
Sedef (47)	Yes	Yes	No	Yes	No	No	No	No	No	Yes	Yes	4/10
Ozgur (48)	Yes	Yes	Yes	Yes	No	No	No	Yes	No	Yes	Yes	6/10
Zhou (51)	Yes	Yes	Yes	Yes	No	No	Yes	No	No	Yes	Yes	6/10
John (16)	Yes	Yes	Yes	Yes	No	No	Yes	Yes	Yes	Yes	Yes	8/10
Richard (50)	Yes	Yes	No	Yes	No	No	No	Yes	Yes	Yes	Yes	6/10
Ayfle (46)	Yes	Yes	Yes	Yes	No	No	Yes	Yes	No	Yes	Yes	7/10
Kong (49)	Yes	Yes	No	No	No	No	No	Yes	No	Yes	Yes	5/10

**Table 3:** GRADE table for effect of LFES on HSP.

Quality assessment							No of patients		Effect	Quality
Outcomes	No of studies	Risk of bias	Inconsistency	Indirectness	Imprecision	Other considerations	LFES	control	Absolute	
Pain intensity	10	Not serious	Serious^a^	Serious^e^	Not serious	None	197	162	SMD −0.68 lower (−1.18 lower to −0.18 lower)	⨁⨁◯ ◯ Low
FMA-UE	4	Not serious	Not serious	Serious^f^	Not serious	None	105	75	MD 8.50 higher (5.12 higher to 11.88 higher)	⨁⨁⨁◯ Moderate

Quality assessment							No of patients		Effect	Quality
Subgroups	No of studies	Risk of bias	Inconsistency	Indirectness	Imprecision	Other considerations	LFES	control	Absolute	
Effect of different stimulation type of LFES on pain intensity in HSP										
TENS	4	Not serious	Serious^a^	Serious^e^	Serious^b^	None	79	64	SMD −0.33 lower (−1.35 lower to 0.69 higher)	⨁◯ ◯ ◯ Very low
NMES	5	Not serious	Not serious	Serious^e^	Not serious	None	105	86	SMD − 0.89 SD lower (−1.28 lower to −0.51 lower)	⨁⨁⨁◯ Moderate
Effect of different stimulation duration of LFES on pain intensity in HSP										
>1 h	3	Not serious	Not serious	Serious^e^	Not serious	None	45	41	SMD −1.16 lower (−1.62 lower to −0.69 lower)	⨁⨁⨁◯ Moderate
≤1 h	7	Not serious	Serious^a^	Serious^e^	Serious^b^	None	152	121	SMD −0.48 lower (−1.10 lower to 0.15 higher)	⨁⨁◯ ◯ Low
Effect of different hemiplegic side of LFES on pain intensity in HSP										
Left hemiparesis ≤ 50%	4	Not serious	Serious^a^	Serious^e^	Serious^b^	None	77	76	SMD −0.58 lower (−1.65 lower to 0.49 higher)	⨁◯ ◯ ◯ Very low
Left hemiparesis > 50%	6	Not serious	Serious^a^	Serious^e^	Not serious	None	120	86	SMD −0.77 lower (−1.24 lower to −0.31 lower)	⨁⨁◯ ◯ Low
Effect of different stimulation frequency of LFES on pain intensity in HSP										
<50 Hz	3	Not serious	not Serious	Serious^e^	Not serious	None	56	39	SMD −0.76 lower (−1.41 lower to −0.10 lower)	⨁⨁⨁◯ Moderate
50–100 Hz	5	Not serious	Serious^a^	Serious^e^	Serious^b^	None	109	94	SMD −0.45 lower (−1.29 lower to 0.38 higher)	⨁◯ ◯ ◯ Very low
Effect of different pulse width of LFES on pain intensity in HSP										
≤200 μs	8	Not serious	Serious^a^	Serious^e^	Not serious	None	173	141	SMD −0.80 lower (−1.30 lower to −0.30 lower)	⨁⨁◯ ◯ Low
>200 μs	2	Serious^c^	Serious^a^	Serious^e^	Very serious^d^	None	24	21	SMD −0.13 lower (−2.00 lower to 1.75 higher)	⨁◯ ◯ ◯ Very low
Effect of different stimulation area of LFES on pain intensity in HSP										
With the trapezius muscle	3	Not serious	Not serious	Serious^e^	Not serious	None	45	41	SMD −1.16 lower (−1.62 lower to −0.69 lower)	⨁⨁⨁◯ Moderate
Without the trapezius muscle	7	Not serious	Serious^a^	Serious^e^	Not serious	None	152	121	SMD −0.48 lower (−1.10 lower to 0.15 higher)	⨁⨁◯ ◯ Low

**Author(s):** Tiantian Hu, Tao Qin, Zhenwei Zhai.

**Question:** Should LFES vs. control group be used for HSP?

**Settings:** Patients come from outpatient clinics or wards, regardless of hospital level.

CI, confidence interval; MD, mean difference; SMD, standardized mean difference.

^a^Evidence of significant heterogeneity; *I*^2^ > 50%.

^b^There is a high level of uncertainty around the effect estimate.

^c^This is a pooled estimate from only two studies.

^d^A very high level of uncertainty around the effect estimate.

^e^Indirect measure of pain intensity.

^f^Indirect measure of upper limb motor function.

### Meta-analysis results

#### Pain intensity

**Figure 4:**
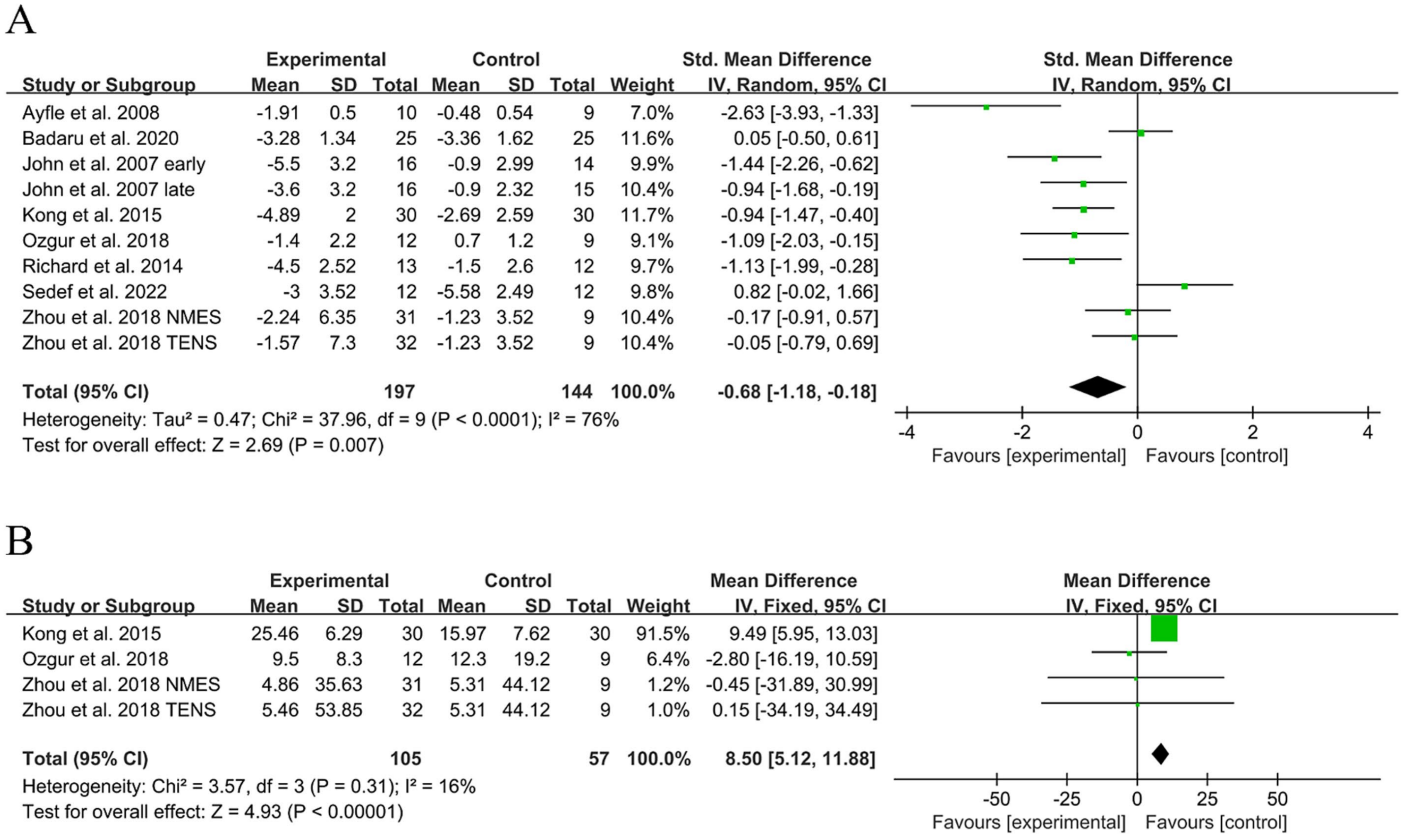
Effect of low-frequency electrical stimulation (LFES) on hemiplegic shoulder pain (HSP). **(A)** Forest plot for pain intensity. **(B)** Forest plot for FMA-UE.

The analysis of pain scale improvement in 341 participants (Figure 4A) revealed the overall effect of LFES. Meta-analysis showed a statistically significant reduction in pain scores in the LFES group compared to the control group (SMD = −0.68; 95% CI: [−1.18, −0.18], *Z* = 2.69, *p* = 0.006, *I*^2^ = 76%). A sensitivity analysis was conducted to assess the impact of study quality, and it was demonstrated that when excluding the two studies (47, 49) with PEDro scores of 4 or 5, the results were not significantly changed (SMD = −0.82; 95% CI: [−1.36, −0.28], *Z* = 2.99, *p* < 0.001; *I*^2^ = 72%).

#### FMA-UE

The overall effectiveness of LFES was assessed by analyzing the improvement on the FMA-UE scale in 180 participants (Figure 4B). Meta-analysis showed that FMA-UE scores were significantly increased in the LFES group compared to the control group (MD = 8.50; 95% CI: [5.12, 11.88], *Z* = 4.93, *p* < 0.001, *I*^2^ = 16%). Additionally, a sensitivity analysis was performed to assess the impact of study quality. We found that excluding a study (49) with PEDro scores of 5 significantly changed the results (MD = −2.14; 95% CI: [−13.74, 9.45], *Z* = 0.36, *p* = 0.72; *I*^2^ = 0%). These results revealed that the overall effect size is particularly sensitive to study quality. This suggests that the observed improvements in motor function from LFES in current studies may require more cautious interpretation.

### Subgroup results

Subgroup analyses were performed to examine whether differences in pain score reduction depended on stimulation type, stimulation duration, hemiplegic side, stimulation frequency, pulse width, and stimulation area. Results for each score were categorized and summarized in terms of SMD and 95% CI and are reported in Supplementary figures and Table 4. Sensitivity analyses were conducted on all subgroups to assess the robustness of the findings, revealing no significant alterations in any subgroup except for the stimulation area and stimulation duration subgroups.

**Table 4:** Effects of LFES on pain scale improvement: subgroup analysis.

Variables	Group (*n*)	Participant (*n*)	MD [95%CI]	*p*	*I*^2^ (%)	Sub-group difference	
						*x^2^*	*p*
Stimulation type							
NMES	5	191	-0.89 [−1.28, −0.51]	<0.001	29%	1.03	0.31
TENS	4	143	−0.33 [−1.35, 0.69]	0.53	84%		
Stimulation duration							
>1 h	3	86	−1.16 [−1.62, −0.69]	<0.001	0%	2.91	0.09
≤1 h	7	255	−0.48 [−1.10, 0.15]	0.13	79%		
Hemiplegic side							
Left hemiparesis ≤ 50%	4	153	−0.58 [−1.65, 0.49]	0.29	88%	0.11	0.74
Left hemiparesis > 50%	6	206	−0.77 [−1.24, −0.31]	0.001	50%		
Stimulation frequency							
<50 Hz	3	95	−0.76 [−1.41, −0.10]	0.02	45%	0.31	0.57
50–100 Hz	5	203	−0.45 [−1.29, 0.38]	0.29	85%		
Pulse width							
≤200 μs	8	314	−0.80 [−1.30, −0.30]	0.002	73%	0.47	0.49
>200 μs	2	45	−0.13 [−2.00, 1.75]	0.90	89%		
Stimulation area							
With the trapezius muscles	3	86	−1.16 [−1.62, −0.69]	<0.001	0%	2.91	0.09
Without the trapezius muscles	7	273	−0.48 [−1.10, 0.15]	0.13	79%		

MD, mean difference; CI, confidence interval.

### Stimulation type

The included studies were stratified into subgroups, as shown in Supplementary Figure S3. No statistically significant differences were observed in the change of pain scores depending on the type of stimulation employed, as there was partial overlap in the 95% CIs of the two groups (*χ*^2^ = 1.03, *p* = 0.31). As four RCTs displayed, the TENS group did not demonstrate a significant advantage in reducing pain scores in HSP patients compared to the control group (SMD = −0.33, 95% CI: [−1.35, 0.69]; *Z* = 0.63, *p* = 0.53; *I*^2^ = 84%). In contrast, RCTs demonstrated superior analgesic efficacy in the NMES group compared with the control group (SMD = −0.89, 95% CI: [−1.28, −0.51]; *Z* = 4.52, *p* < 0.001, *I*^2^ = 29%).

### Stimulation duration

The included studies in this meta-analysis were stratified into two subgroups based on stimulation duration, as illustrated in Supplementary Figure S4. No statistically significant differences were found concerning changes in pain scores (*χ*^2^ = 2.91, *p* = 0.09). However, findings from three RCTs demonstrated that the LFES group with a stimulation duration >1 h/day exhibited superior efficacy in reducing HSP, leading to significant improvements in pain scores compared to the control group (SMD −1.16, 95% CI: [−1.62, −0.69]; *p* < 0.001; *I*^2^ = 0%). Conversely, the remaining seven RCTs revealed no statistically significant advantage in terms of the analgesic effect within the LFES stimulation duration ≤1 h/day group when compared to the control group. (SMD −0.48, 95% CI: [−1.10, 0.15]; *Z* = 1.50, *p* = 0.13; *I*^2^ = 79%). The sensitivity analysis revealed that excluding the study conducted by Sedef et al. (47), had a significant impact on the results of the treatment duration ≤1 h/day group (*p* = 0.03), and the heterogeneity was reduced (*I*^2^ = 75%).

### Hemiplegic side

When studies were grouped by hemiplegia side (Supplementary Figure S5), significant pain score reduction was observed in the LFES group compared to the control group in studies with the left side as the predominant side of hemiplegia (SMD −0.77, 95% CI: [−1.24, −0.31]; *Z* = 3.27, *p* = 0.001; *I*^2^ = 50%). In studies where the right side was the predominant hemiplegic side, there was no statistical difference in pain improvement between the LFES and control groups (SMD −0.58, 95% CI: [−1.65, 0.49]; *Z* = 1.06, *p* = 0.29; *I*^2^ = 77%). However, no differences in change in pain scores were observed in studies with left hemiplegia >50% or left hemiplegia ≤ 50% (*χ*^2^ = 0.11, *p* = 0.74).

### Stimulation frequency

When studies were grouped by stimulation frequency (Supplementary Figure S6), significant improvement in pain scores was observed for studies with LFES frequency <50 Hz compared to the control group (SMD −1.41, 95% CI: [−1.41, −0.10]; *Z* = 2.27, *p* = 0.02; *I*^2^ = 0%). Five trials revealed that the administration of LFES at 50–100 Hz did not yield a statistically significant difference in pain relief when compared to control groups (SMD −0.45, 95% CI: [−1.29, 0.38]; *Z* = 1.06, *p* = 0.29; *I*^2^ = 85%). However, there was no statistically significant difference observed in pain scores change between studies that employed LFES frequencies <50 Hz and those utilizing frequencies at 50–100 Hz (*χ*^2^ = 0.31, *p* = 0.57).

### Pulse width

A subgroup analysis was conducted based on pulse width to evaluate the effect of pain improvement, as presented in Supplementary Figure S7. The analysis revealed no statistically significant differences in the change of pain scores (*χ*^2^ = 0.47, *p* = 0.49). However, among the included studies, eight RCTs demonstrated that LFES with a pulse width ≤200 μs showed superiority compared to control groups (SMD −0.80, 95% CI: [−1.30, −0.30]; *Z* = 3.13, *p* = 0.002; *I*^2^ = 74%). Two RCTs indicated no statistically significant difference in pain improvement between the administration of LFES with a pulse width >200 μs and control groups (SMD −0.13, 95% CI: [−2.00, 1.75]; *Z* = 0.13, *p* = 0.95; *I*^2^ = 89%).

### Stimulation area

A subgroup analysis of pain improvement was performed. We stratified the included studies according to subgroups, as shown in Supplementary Figure S8. Both subgroups demonstrated the superiority of the LFES group over the control group (*χ*^2^ = 2.91, *p* = 0.09). Three RCTs revealed the dominant effect of LFES with the trapezius muscle as the stimulation area compared to the control group (SMD −1.16, 95% CI: [−1.62, −0.69]; *Z* = 4.89, *p* < 0.001; *I*^2^ = 0%). Seven trials revealed there was no the superiority of LFES without the trapezius muscle as the stimulation area over the control group (SMD − 0.48, 95% CI: [−1.10, 0.15]; *Z* = 1.50, *p* = 0.13; *I*^2^ = 79%). The sensitivity analysis revealed that the exclusion of the data from Sedef et al. (47) had a notable impact on the results of the LFES without the trapezius muscle group (*p* = 0.03), while the level of heterogeneity remained largely unaffected.

## Discussion

This meta-analysis evaluated the effectiveness of LFES compared to other monotherapies, placebo/sham treatments, or conventional rehabilitation in reducing HSP in stroke survivors. The results showed a statistically significant effect, indicating an overall reduction in pain intensity after LFES treatment. However, regarding motor function scores, although some measures improved, the findings for the FMA-UE scale should be interpreted cautiously, as sensitivity analysis did not robustly support its efficacy. Sensitivity analysis revealed that the pooled effect size was disproportionately influenced by a single study of fair methodological quality (Kong et al.), which exhibited limitations such as inadequate allocation concealment and lack of blinding. The inclusion of this study likely led to an overestimation of LFES efficacy on motor function, despite the low overall heterogeneity (*I*^2^ = 16%). When this study was excluded, the effect size was notably reduced (MD = 5.12; 95% CI: [2.15, 8.09]), underscoring the need for cautious interpretation of the findings. Consequently, these findings warrant verification through future large-scale RCTs employing standardized FMA-UE assessments and rigorous design. Subgroup analyses conducted to evaluate the effectiveness of LFES in reducing pain scores in patients with HSP did not reveal significant differences between subgroups based on specific factors such as stimulation type, hemiplegic side, stimulation frequency, and pulse width. However, upon conducting subgroup analysis based on stimulation area and stimulation duration, statistically significant differences in reductions of pain scores were observed between the respective subgroups.

The efficacy of ES to mitigate shoulder pain in hemiplegic patients has been controversial in previous meta-analyses. While certain studies propose potential advantages of ES in mitigating shoulder subluxation and enhancing range of motion, the comprehensive evidence concerning its direct efficacy in alleviating shoulder pain exhibits different findings (28–30). Notably, three of these reviews concluded that ES does not significantly alleviate pain in patients with HSP. However, it is important to note that these studies often included participants who had experienced a stroke but did not necessarily present with shoulder pain at baseline, which may have affected the pooled results on ES’s efficacy for HSP. Only one meta-analysis reported a significant benefit of ES in improving HSP, suggesting that there may be unexplored factors contributing to its efficacy. Recognizing these limitations, our review expands upon the previous literature in several critical ways. Firstly, more RCTs (eight) with a wider range of languages (English, Turkish, Nigerian, and Chinese) and participants (341) were included in our study. Secondly, considering the different treatment mechanisms in different frequencies of ES, only LFES was analyzed in our study to avoid latent bias. We notice that some review articles clearly indicate that a single type of LFES has a certain therapeutic effect on HSP (29, 52), but a comprehensive comparison of more types of LFES has not been fully considered. Our study offers a comprehensive analysis of LFES for treating HSP, aiming to elucidate the optimal treatment parameters and modalities.

The etiopathogenesis of HSP remains complex and not yet fully elucidated. Musculoskeletal and neurological factors are primarily responsible for this complexity. Current research predominantly focuses on musculoskeletal factors such as shoulder subluxation, spasticity, muscle imbalance, and structural impairment (13, 14, 53). RCTs demonstrated that NMES applied to the supraspinatus and posterior deltoid significantly decreased shoulder subluxation and pain while improving muscle strength (54). For spasmodic muscles, ES relieves excessive muscle tension and relaxes muscles (55). On the other hand, neurological factors including sensory alteration, neuropathic pain mechanism, and central sensitization also emerge as noteworthy factors (15). Studies have shown that ES can relieve pain by blocking pain signaling (56). Additionally, inflammation, endothelial dysfunction, and immune system deficiencies may potentially contribute to the pathogenesis of complex regional pain syndrome (CRPS), thereby giving rise to HSP (57, 58). Understanding the intricate etiopathogenesis contributes to developing effective therapeutic interventions. The effect of LFES on improving HSP may be influenced by various factors, including stimulation type, stimulation frequency, pulse width, stimulation duration, hemiplegic side, and stimulation area. These parameters can potentially play a significant role in determining the effectiveness of LFES as a therapeutic intervention for HSP.

### Stimulation type

Currently, the stimulation type of LFES primarily includes TENS and NMES. Previous studies showed that both TENS and NMES are effective for improving HSP (51). TENS is thought to reduce pain by delivering a low-voltage electrical current through electrodes placed on the skin. This technique is believed to selectively activate larger diameter sensory C-fibers as opposed to motor fibers, thereby contributing to relieving pain (59). The precise control of sensory nerve stimulation in this way prevents patients from experiencing discomfort or spasms in their motor muscles during the treatment, making TENS an effective and relatively safe method for pain management (60, 61). Mainly involves Pain Gate Mechanisms and the Endogenous Opioid System (62, 63). Specifically, TENS exerts its effects by selectively stimulating peripheral sensory fibers and activating the dorsal horn glial cells to close the pain gate, thereby reducing central excitability and reducing pain. Furthermore, TENS has the potential to activate specific receptors such as opioids, GABA, serotonin, acetylcholine, and cannabinoid receptors to activate endogenous inhibitory mechanisms in the central nervous system (CNS) and produce analgesic effects (64–66). Additionally, TENS may produce analgesic effects through peripheral mechanisms. Firstly, it may decrease the release of substance P in dorsal root neurons, consequently mitigating tissue damage (67). On the one hand, it may stimulate sensory receptors in the skin, thereby leading to axonal reflexes or regeneration, and improving local circulation (68). In addition, TENS may reduce the production of pain-related neuropeptides by modulating NF-κB complex, toll-like receptor-7, and phosphoinositide 3-kinase/Akt signaling pathways (69). These mechanisms indicate that TENS mainly plays a role in the treatment of neuropathic pain.

In contrast to TENS, NMES is commonly used for muscle strengthening, preventing muscle atrophy, enhancing joint range of motion, and facilitating muscle healing and recuperation. Studies have demonstrated the therapeutic potential of NMES in managing various conditions, including patellofemoral pain syndrome, knee osteoarthritis pain, low back pain, and HSP (70–74). Nevertheless, the mechanism underlying the analgesic effect of NMES remains insufficiently investigated. Based on the role of NMES in neuromuscular control, the principle of NMES in the treatment of pain may be realized through the following aspects. First, NMES-triggered muscle contractions engender a muscle pump effect, thereby ameliorating blood perfusion and lymphatic drainage within the targeted region (75). This augmentation in circulatory dynamics expedites the elimination of inflammatory mediators and metabolic byproducts, including prostaglandins and leukotrienes, which are pivotal contributors to the genesis of nociceptive sensations during the inflammatory cascade (76). Furthermore, extensive research has demonstrated the significant therapeutic effect of NMES in ameliorating spasms arising from various CNS disorders, including cerebral palsy (77) and stroke (78). It is worth noting that muscle spasms often engender pain through mechanisms involving muscular hypoxia, nerve compression, and localized inflammatory responses (79, 80). The precise underlying mechanism of NMES to ameliorate spasticity remains elusive, yet previous investigations have provided evidence that NMES can modulate spinal reflexes and influence the excitability of spinal circuits (81–83). Moreover, NMES has been found to regulate CNS plasticity, thereby offering relief from spasms. Consequently, it can be postulated that NMES may potentially alleviate pain associated with spasms.

Despite the comparisons made in existing RCTs regarding the effectiveness of NMES and TENS in the context of HSP, there is currently a lack of higher-level evidence that definitively establishes which type of LFES is more suitable for HSP treatment. Consequently, our report fills this gap by conducting a novel subgroup analysis based on the LFES stimulation type, an area that has not been extensively explored in previous meta-analyses. In our results, we found that NMES demonstrated a statistically significant improvement in pain scores compared to the control group, whereas TENS did not show a significant reduction in pain scores. This conclusion aligns with previous research, which has consistently demonstrated that although both TENS and NMES are effective in improving HSP, NMES surpasses TENS in sustaining long-term analgesia (51). Another single-blind, two-arm RCTs showed that EMG-triggered NMES with bilateral arm training demonstrated greater immediate and retained effects than TENS with bilateral arm training on pain score improvement in chronic and subacute stroke patients with HSP (70). There are many possible explanations for our results. A putative explanation for these results could be the majority of cases of HSP could be attributed to musculoskeletal factors. NMES enhances muscle strength, endurance, and coordination to mitigate shoulder subluxation (84) and muscle imbalances (85–87). Muscle imbalance may increase biomechanical strain on the shoulder joint, resulting in disrupted blood flow, nerve entrapment, and inflammation, exacerbating discomfort (88, 89). Another explanation is that NMES offers deeper stimulation than TENS, effectively targeting underlying musculoskeletal issues in hemiplegic patients by reaching both superficial muscles and deeper structures such as joint capsules and tendons, thereby restoring function and reducing pain (51, 90–92). This modality aids in restoring normal function and alleviating pain in these structures. Although the superior effectiveness of NMES is suggested, it is crucial to interpret these findings within the context of the overlapping CIs between the two stimulation subgroups. Our report fills the gap by conducting a novel subgroup analysis based on the LFES stimulation type, an area not extensively explored in previous meta-analyses.

### Stimulation duration

Previous studies have reported that prolonged stimulation induces adaptive changes in the nervous system, leading to a more sustained analgesic effect (93, 94). Prolonged stimulation elicits a broader range of physiological responses, including neuromuscular remodeling, improved blood circulation, and immune system regulation, which may contribute positively to pain relief (95, 96). Furthermore, long-term stimulation has been shown to promote tissue repair and regeneration processes, thereby expediting pain relief. Enhanced blood flow facilitates the delivery of oxygen and nutrients to tissues while removing toxins and reducing inflammation, resulting in pain reduction (97). Increased blood flow also alleviates muscle pain caused by low oxygenation levels and poor circulation through improved muscle oxygenation (98, 99). Studies have revealed that ES can enhance immune cell proliferation, cytokine secretion, extracellular matrix production, and vascular development involving macrophages as well as B cells and T cells (100, 101). Electric field application induces macrophage polarization into distinct subtypes and modulates T cell migration, proliferation, and cytokine production; thus highlighting the potential of ES in modulating immune responses (102). Additionally, long-term stimulation can modulate neural pathways associated with pain perception. Chronic pain conditions often involve aberrant CNS mechanisms. Prolonged ES may regulate these pathways and reduce central sensitization and pain perception (103). Long-term potentiation (LTP) involves such a process that more effective signals transition through synapses between neurons, typically associated with the functions of learning and memory (104). Long-term stimulation can induce LTP-like changes in the nervous system, leading to sustained improvements in pain perception and tolerance (105). Prolonging treatment duration may induce the structural changes including synaptic connections and neural plasticity in the nervous system, leading to durable improvements in pain management (106).

Our subgroup analysis demonstrated that in patients with HSP, a noteworthy improvement in pain scores is observed when LFES is administered for a duration >1 h. This implies that the achievement of optimal outcomes with LFES may necessitate a prolonged stimulation period. Nevertheless, it should be noted that previous studies have reported an elevated incidence of adverse events associated with prolonged ES therapy. These adverse events may encompass musculoskeletal pain, erythema, dermatitis, burns, and other potential complications (107, 108). The review of the included literature revealed that a majority of the studies implemented a stimulation duration ranging from 30 to 60 min. This practice may have been influenced by factors such as concerns over adverse reactions associated with prolonged stimulation, patients’ subjective comfort and tolerance, and the potential impact of sustained stimulation on neural adaptation (109, 110). However, it is important to note that there currently exist no established guidelines providing specific recommendations or guidance regarding the optimal total duration or frequency of LFES treatment. Notably, findings from an animal experiment indicated that in comparison to shorter continuous stimulation, longer intermittent stimulation is less likely to induce selective activation of nociceptive neurons, muscle damage, and the release of endogenous opioid drugs (111). This evidence suggests that the integration of long-term intermittent stimulation may offer a promising approach to strike a balance between treatment efficacy and the occurrence of adverse events. Nonetheless, additional research studies or the development of comprehensive clinical guidelines are warranted to ascertain the optimal treatment duration for diverse patient populations and varying clinical scenarios. Such investigations would contribute valuable insights into tailoring treatment protocols and enhancing therapeutic outcomes while minimizing potential risks.

The articles we included showed that all the treatments of stimulation duration (>1 h) were implantable (16, 50). The implantable peripheral nerve ES usually lasts for long periods (less than 6 h). In light of safety concerns pertaining to continuous and protracted stimulation, it is common practice to administer this form of stimulation intermittently as opposed to continuously (112–114). Clara Gunter et al. have summarized the safety parameters of peripheral nerve ES under a long treatment period, which highlighted that less than 50% of the effective stimulation time is considered safe (95). Implantable devices facilitate the direct and precise stimulation of specific nerves or neural structures, offering distinct advantages over non-implantable ES (115). This targeted stimulation has the capacity to modulate neural activity more effectively, resulting in enhanced therapeutic outcomes for a range of neurological and pain-related conditions (116). However, even though the intermittency and duration of ES are well controlled, tissue damage and chronic inflammation caused by electrodes implanted surgery cannot be ignored (107, 117). The foreign body reaction in electrode implantation can cause chronic inflammation and capsule, impairing the long-term ability and durability (108). Strategies to reduce foreign body reaction include improving the material properties of the electrodes, providing anti-inflammatory drugs, minimizing neuronal loss, promoting nerve regeneration, and limiting the formation of glial sheaths (107, 111). Although implantable ES devices hold promise for pain management in specific scenarios, the relative importance of stimulation duration and mode remains unclear. Thus, future research should explore the potential impact of one factor on the overall effect while controlling for the other.

### Hemiplegic side

Studies have shown that left hemiplegia, which is associated with right hemispheric stroke, is a significant risk factor for HSP (118). This elevated risk may be attributable to a constellation of factors associated with right hemispheric lesions. For instance, the right hemisphere plays a critical role in spatial awareness, attention, and the integration of visual–spatial information. Damage to this area can result in hemispatial neglect, impaired proprioceptive feedback, and difficulties in recognizing and responding to stimuli on the affected side, all of which may contribute to malalignment, altered muscle activation patterns, and an increased susceptibility to shoulder pain (119, 120). Building on these observations, we hypothesize that patients with left HSP secondary to right hemisphere stroke may experience more pronounced disruptions in sensorimotor processing and pain perception due to the hemisphere’s role in integrating sensory input and guiding motor responses (121). The severity of neglect and spatial-processing deficits could reduce patients’ ability to properly stabilize and move the shoulder, thereby exacerbating pain and mechanical strain. Therefore, the therapeutic effect of LFES on such patients could be influenced by anatomical and physiological factors. Surprisingly, our results indicated that LFES may be more effective in alleviating shoulder pain in patients with left hemiplegia. One possible explanation for this finding is that LFES, as a peripheral intervention, enhances sensory and motor feedback to the CNS, promoting CNS plasticity and facilitating brain function remodeling (122, 123). Additionally, LFES may improve the awareness and attention of patients with left-sided neglect toward their affected limb, encouraging active participation in rehabilitation and reducing compensatory movements that could exacerbate shoulder pain (124, 125). Given these findings, early initiation of LFES therapy may be crucial for effectively managing HSP in patients with left hemiplegia, particularly because right hemispheric damage may lead to more pronounced challenges in motor and sensory processing.

### Stimulation frequency

As is known to all, the specific effect of LFES is affected by various factors, such as treatment time, treatment intensity, and individual differences. Different frequency parameters are crucial for regulating effects on neural function and physiological processes. Considering pain mechanisms, LFES between 1 and 5 Hz frequencies stimulate the release of neuropeptides, such as substance P and calcitonin gene-related peptide (CGRP). These peptides regulate nociceptive processes and relieve pain by affecting neuronal excitability and synaptic transmission (126). LFES with frequencies between 5 and 10 Hz induces the release of endogenous opioids, such as endorphins and enkephalins. These neurotransmitters bind to opioid receptors in the CNS, exerting analgesic effects and reducing pain perception (126). LFES with frequencies between 10 and 20 Hz regulates central sensitization, which exhibits an increase in the excitability of central nociceptive neurons, leading to hyperalgesia and abnormal pain. By inhibiting central sensitization, LFES with frequencies within this range helps to relieve hyperalgesia and abnormal pain (127, 128). LFES with frequencies between 20 and 50 Hz triggers descending inhibitory pathways from the brainstem to the spinal cord, promoting the release of neurotransmitters such as serotonin and norepinephrine, thereby regulating the transmission of pain signals (129). Higher frequency LFES above 50 Hz inhibits the transmission of pain signals carried by smaller diameter Aδ and C fibers by activating large diameter Aβ nerve fibers, which is controlled by synaptic inhibition in layer II of dorsal horn neurons. This process can cover or “close” pain stimuli by closing the neural gate in the spinal cord, thereby reducing pain perception (129, 130). Theoretically, higher frequency ES may play a more effective analgesic effect due to its effect on pain gating mechanisms. However, our study found that LFES significantly improved the pain score of HSP with a stimulation frequency of <50 Hz compared with the control group. Moreover, the improvement of pain score was more significant compared with LFES with a frequency range of 50–100 Hz. This may be related to the musculoskeletal pathogenesis of HSP. The optimal stimulation frequency of human skeletal muscle cells is 30–80 Hz (131, 132). LFES often causes skeletal muscle cells to produce a single contraction, which gradually changes from a single contraction to a tonic contraction with an increase in frequency. Although the strength of muscle contraction increases with the increase of frequency, excessive frequency stimulation may lead to rapid muscle fatigue and decreased muscle endurance, thus failing to play the optimal therapeutic effect (133). On the other hand, ES at different frequencies recruits muscle fibers with different properties. Lower frequencies (e.g., 30–50 Hz) primarily activate slow muscle fibers, which are important for endurance and sustained contraction. Higher frequencies (e.g., 50–80 Hz) are more effective in recruiting rapidly twitching muscle fibers, which produce rapid and forceful contraction required for strength and power activities (134). Further studies are needed to fully elucidate the underlying mechanisms driving these differential effects of LFES frequencies on pain relief.

### Pulse width

Pulse width impacts motor neuron recruitment and patient comfort. LFES with a pulse width ≤200 μs significantly reduced HSP pain scores in our study. Yet, no notable difference was found between higher pulse width (>200 μs) LFES and control groups. Limited meta-analysis exists on LFES efficacy regarding pulse width settings for HSP. Our findings imply that LFES with lower pulse width (≤200 μs) could benefit HSP treatment more. Therefore, a potential explanation is that by using lower pulse widths, LFES can selectively activate the necessary nerve fibers associated with pain relief and muscle function while minimizing the activation of unnecessary neural pathways (135, 136) This targeted method addresses musculoskeletal factors like muscle weakness, spasticity, or altered activation patterns causing shoulder pain in hemiparetic individuals. Studies indicate effective movement stimulation occurs within 20–200 μs, sans pain responses (137).

### Stimulation area

The stimulation area for LFES in our studies primarily targeted the supraspinatus muscle and posterior deltoid, both crucial for shoulder stability and movement and common targets for ES due to their superficial location (138, 139). The specific placement of the adhesive electrodes was not described in the two articles included in our study (46, 47). In contrast, two articles emphasized that the stimulation area included the trapezius muscle (16, 50). The trapezius muscle, crucial for shoulder stability, significantly influences scapular movement and overall shoulder function, facilitating daily activities like lateral arm raising and scapula retraction (140, 141). Our analysis indicated that pain scores significantly improved when the stimulation area included the trapezius muscle. In contrast, no significant improvement in pain scores was observed when the stimulation area excluded the trapezius muscle. Superior outcomes in the trapezius stimulation subgroup may be attributed to broader muscle activation, which potentially addresses the imbalance and weakness underlying shoulder pain in individuals with hemiplegia.

### Study limitations

It is important to interpret these results cautiously due to certain limitations. First and foremost, the limited number of included RCTs (*n* = 8) must be emphasized as a critical limitation. Despite conducting a comprehensive, unrestricted literature search across seven major databases, only a small pool of eligible studies was identified. This paucity of high-quality RCTs in the field may restrict the statistical power and generalizability of our findings. Second, it should be noted that all the studies included in our analysis had relatively small sample sizes (*n* = 19–61), which could potentially lead to an overestimation of the estimated effect size (142). The interpretation of motor function (FMA-UE) outcomes should be made cautiously, as the sensitivity analysis identified two studies with suboptimal methodological quality that disproportionately affected the meta-analysis results. Given the scarcity of included studies reporting FMA-UE (*n* = 4), future large-scale RCTs employing standardized, high-quality designs are warranted to validate these preliminary findings. Third, only one study reported information on adverse events, limiting our understanding of the potential harms of LFES in patients with HSP. Fourth, sensitivity analysis showed that the primary outcome measures were not significantly affected. However, excluding the study by Kong et al. (49) significantly changed the secondary outcome measures. Examination revealed that Kong et al.’s effect size was significantly higher than that of other studies, possibly due to their use of the nested VAS pain intensity rating from the McGill Pain Questionnaire without detailed evaluation criteria. Fifth, another major limitation is the quality of the included studies. Three studies lacked adequate allocation concealment. Because LFES causes noticeable changes and requires parameter adjustments, none of the studies reported patient or researcher blinding. Additionally, six studies lacked long-term follow-up evaluations and intention-to-treat data. The lack of blinding and long-term evaluation may overestimate effects and limit external validity (143). Although we conducted a comprehensive search across multiple databases, our review did not include gray literature such as unpublished dissertations, conference abstracts, trial registries, and other non-indexed sources. This may introduce potential publication bias, as negative or neutral results are often underrepresented in peer-reviewed journals. However, both funnel plot inspection and statistical tests (Egger’s test and Begg’s test for pain outcomes) did not detect significant asymmetry, suggesting a low likelihood of substantial publication bias. Nevertheless, caution is warranted when interpreting the findings, as undetected bias may still exist due to limitations in gray literature retrieval.

Despite these limitations, this current meta-analysis provides evidence-based insights into the use of LFES for the treatment of HSP and offers valuable guidance for the clinical application of stimulation parameters and treatment protocols. Additionally, previous research on HSP does not classify pain types, resulting in limited exploration of stimulation parameters (e.g., frequency, duration, pulse width) for different pain types. Future studies should investigate the differential efficacy of various parameters for different types of pain, such as musculoskeletal pain versus neuropathic pain. Moreover, future systematic reviews and meta-analyses should expand their search scope to include credible conference proceedings, dissertations, and other gray literature supported by clinical data to maximize the comprehensiveness of the evidence base and reduce the risk of bias.

## Conclusion

Our systematic analysis suggests that LFES may have beneficial effects on pain scores in stroke patients with HSP, though improvements in motor function remain less conclusive. The current evidence, while promising, is limited by study quality and sample sizes. LFES appears to be a potentially promising intervention for HSP, particularly when applying specific parameters (stimulation duration >1 h, stimulation frequency ≤50 Hz, pulse width ≤200 μs, trapezius muscle inclusion). However, these observations require confirmation through rigorously designed, large-scale RCTs with standardized protocols and long-term follow-up. Therefore, LFES appears to be a promising approach for alleviating hemiplegic shoulder pain; however, given the limited number of high-quality studies and variability in stimulation parameters, further rigorously designed randomized controlled trials are needed to confirm its efficacy and optimize treatment protocols.

## Data Availability

The original contributions presented in the study are included in the article/Supplementary material, further inquiries can be directed to the corresponding author.

## Footnotes

^1^ www.pedro.org.au

## Glossary

CI: confidence interval
CNKI: China National Knowledge Infrastructure
CNS: central nervous system
CRPS: complex regional pain syndrome
EMBASE: Excerpta Medica Database
ES: electrical stimulation
FES: functional electrical stimulation
FMA-UE: Fugl-Meyer assessment for upper extremities
GRADE: Grading of Recommendations Assessment, Development and Evaluation
HSP: Hemiplegic shoulder pain
LFES: low-frequency electrical stimulation
LTP: long-term potentiation
NMES: neuromuscular electrical stimulation
NRS: numerical rating scale
PEDro: Physiotherapy Evidence Database Scale
PNS: peripheral nerve stimulation
PRISMA: Preferred Reporting Items for Systematic Reviews and Meta-analysis
RCTs: randomized controlled trials
RevMan: Review Manager
RoB 2: Version 2 of the Cochrane Risk of Bias tool
SMD: standardized mean difference
TENS: transcutaneous electrical nerve stimulation
VAS: visual analog scale
VIP: China Science and Technology Journal Database
WMD: weighted mean difference
